# Long-acting injectable antipsychotics during pregnancy: An update

**DOI:** 10.1192/j.eurpsy.2021.2204

**Published:** 2021-08-13

**Authors:** A. Paraschakis, M. Papasaika

**Affiliations:** Psychiatric Hospital Of Attica “dafni”, Department of General Adult Psychiatry, Athens, Greece

**Keywords:** Long-acting injectable antipsychotics, schizophrénia, bipolar disorder, pregnancy

## Abstract

**Introduction:**

Long-acting injectable (LAI) antipsychotics are related to proven compliance to treatment and more constant medication levels (hence the apparent lower side-effect burden).

**Objectives:**

To highlight the experience with LAI antipsychotic treatment during pregnancy.

**Methods:**

Literature review.

**Results:**

Seven cases are reported. A 35year old with schizophrenia received zuclopenthixole LAI (mostly 200mg/monthly) during both her pregnancies (of healthy girls born at weeks 39 and 40). A 35year old with schizophrenia was under risperidone LAI (25mg/2 weeks) and gave birth to a healthy girl at week 37. Another 35year old (probably with schizophrenia) was on olanzapine LAI (300mg/month during the last quarter of her pregnancy) that led to the birth of a healthy girl at week 40. A 37year old with schizophrenia received paliperidone LAI (100mg/monthly, last injection at week 28) and gave birth to a healthy boy at week 39. Paliperidone LAI (50mg/monthly) was the treatment of another 34year old with schizoaffective disorder that gave birth to a healthy boy at week 40, as well as of a 26year old (263mg/3-monthly), mother of a healthy boy as well (born at an unspecified week of pregnancy). Finally, a 43year old with bipolar disorder was on aripiprazole LAI (300mg/monthly) during her pregnancy that led to the birth of a healthy girl at week 40.

**Conclusions:**

All pregnant women on LAI antipsychotic treatment gave birth to (apparently) healthy babies. LAI doses were mostly low. Long-term follow-up could clarify eventual delayed aftereffects. Based on the literature, LAI antipsychotic treatment could be considered as an option for selected pregnant patients.

**Disclosure:**

No significant relationships.

